# Chemical and physical factors of desensitizing and/or anti-erosive toothpastes associated with lower erosive tooth wear

**DOI:** 10.1038/s41598-017-18154-8

**Published:** 2017-12-20

**Authors:** Samira Helena João-Souza, Adrian Lussi, Tommy Baumann, Taís Scaramucci, Ana Cecília Corrêa Aranha, Thiago Saads Carvalho

**Affiliations:** 1 Department of Preventive, Restorative and Pediatric Dentistry, Labor C331, University of Bern, Freiburgstrasse 7, CH-3010 Bern, Switzerland; 2 Department of Restorative Dentistry, University of São Paulo, School of Dentistry. Av. Prof. Lineu Prestes 2227, Cidade Universitária, São Paulo, SP 05508-000 Brazil

## Abstract

Toothpastes have a complex formulation and their different chemical and physical factors will influence their effectiveness against erosive tooth wear (ETW). We, therefore, investigated the effect of different desensitizing and/or anti-erosive toothpastes on initial enamel erosion and abrasion, and analysed how the interplay of their chemical and physical factors influences ETW. Human enamel specimens were submitted to 5 erosion-abrasion cycles using 9 different toothpastes and an artificial saliva group, and enamel surface loss (SL) was calculated. Chemical and physical factors (pH; presence of tin; calcium, phosphate and fluoride concentrations; % weight of solid particles; wettability; and particle size) of the toothpaste slurries were then analysed and associated with the amount of SL in a multivariate model. We observed that all desensitizing and/or anti-erosive toothpastes presented different degrees of SL. Besides pH and fluoride, all other chemical and physical factors were associated with SL. The results of this experiment indicate that enamel SL occurs independent of whether the toothpastes have a desensitizing or anti-erosive claim, and that lower SL is associated with the presence of tin, higher concentration of calcium and phosphate, higher % weight of solid particles, smaller particle size, and lower wettability.

## Introduction

Erosive tooth wear (ETW) results from the contact of erosive substances with the dental surfaces, in association with mechanical forces, such as toothbrush abrasion, tongue and cheek movements^1^. The continuous incidence of erosive and abrasive activities on the enamel surface can expose the underlying dentine, leaving open tubules, which can cause a short and sharp pain defined as dentine hypersensitivity^2,3^. Many efforts have been made to protect the enamel surface and prevent the progression of ETW. Among the products available over-the-counter, toothpaste is the most common oral care product used by the population.

Nowadays, toothpastes claim to have a multi care action and/or to have specific goals, such as desensitizing and/or anti-erosive properties. Regardless of their claim, toothpastes should protect the teeth, preventing enamel surface loss. Toothpastes with an anti-erosive claim have been tested, and they did not show a superior protective effect against ETW when compared to conventional fluoridated toothpastes^4^. Thus, it is important to analyse the effect of different toothpastes on enamel erosion-abrasion, particularly regarding the complex formulations of these toothpastes. Toothpaste formulation can influence the effectiveness of their active ingredients, especially under abrasive conditions, where the efficacy of the active ingredients is not always as expected. The solid particles (abrasives) play a big role in the effect of toothpastes against ETW^4–6^. The abrasives may either react with the active ingredients, hampering the viability and effect of the latter, or increase enamel surface loss due to a greater mechanical action in association with the toothbrush.

In view of the wide range of new active ingredients and amounts of abrasives used in toothpastes, there is a need for more studies regarding the interplay between ETW and the chemical and physical factors of these toothpastes. This *in vitro* study aimed at investigating the effect of desensitizing and/or anti-erosive toothpastes on initial enamel erosion and abrasion, and analyse how the chemical and physical factors of these toothpastes influence ETW. The null hypotheses were: 1) there is no difference between the toothpastes in enamel protection regarding their claim; 2) none of the toothpastes are able to protect enamel from erosion and abrasion; and 3) chemical and physical factors of the toothpastes are not associated with enamel surface loss.

## Results

**Figure 1:**
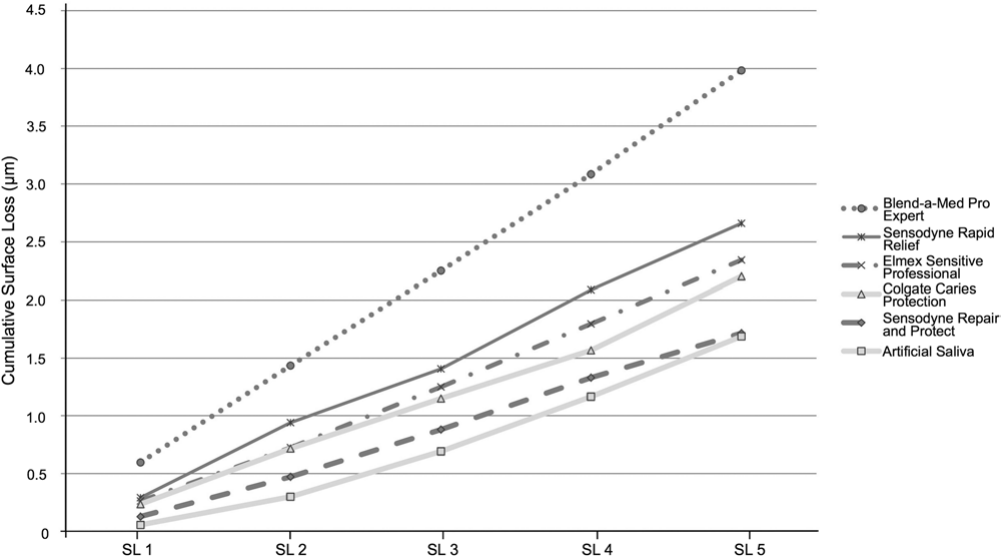
Enamel surface loss (SL) after each cycle, for desensitizing toothpastes (light grey lines represent the control groups).

**Figure 2:**
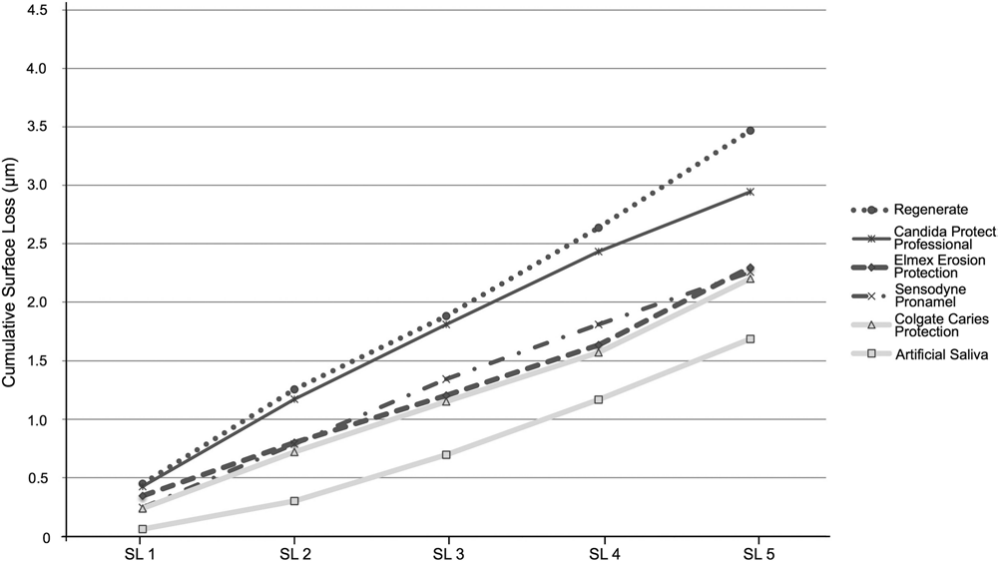
Enamel surface loss (SL) after each cycle, for anti-erosive toothpastes (light grey lines represent the control groups).

**Figure 3:**
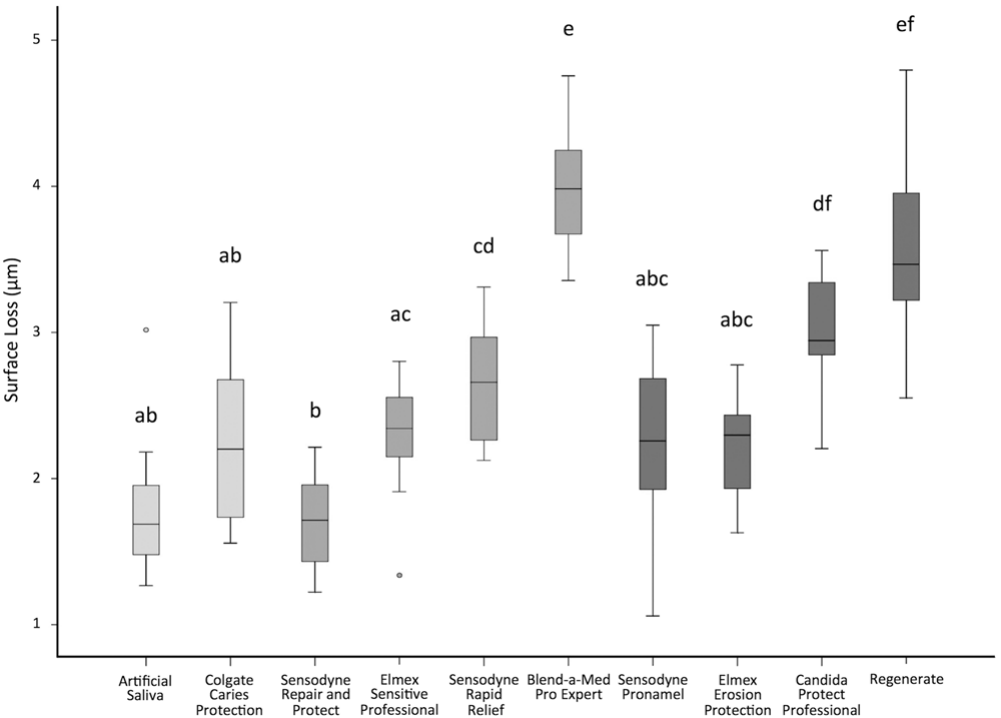
Total enamel surface loss after 5 cycles, for the different experimental groups, according to the claim of the toothpaste: light grey boxes – control groups, medium grey boxes – desensitizing claim; dark grey boxes – anti-erosive claim. Different letters denote significant differences between the groups.

All groups showed progressive SL throughout the experiment (Figs 1 and 2). After 5 cycles, the toothpastes presented different amounts of SL (Fig. 3). Among the desensitizing toothpastes, Sensodyne Repair and Protect showed a numerically lower SL value, but it was not different from both control groups (AS and Colgate Caries Protection, p > 0.05). Blend-a-Med Pro Expert showed the highest SL. Regarding the anti-erosive toothpastes, Sensodyne Pronamel and Elmex Erosion Protection presented the lowest values, not differing from the control groups and with no difference between them (p > 0.05), and Regenerate showed the highest SL values, which were not different from Blend-a-Med Pro Expert (p > 0.05). The amount of SL was not associated with the claim of the toothpastes, as confirmed by a bivariate regression (Estimate ± Standard Error: −0.110 ± 0.144; p = 0.444).

Table 1 presents the chemical and physical factors in AS and toothpaste slurries. AS presented neutral pH (7.0), no solid particles, and concentration of Ca^2+^, PO_4_^3−^ and free F^−^ of 0.59 mmol/l, 8.5 × 10^−3^ µmol/l and 0.07 ppm, respectively. Regarding the toothpastes, the pH ranged from 4.7 to 9.0; all presented small particle sizes (<20 µm), but Sensodyne Rapid Relief, Blend-a-Med Pro Expert and Regenerate had particles ≥50 µm. The amount of solid particles ranged from 25 − 41%, and the ion concentrations ranged: for Ca^2+^ between 0.08 − 8.90 mmol/l; PO_4_^3−^ between 8.4 × 10^−8^ − 66.05 µmol/l; free F^−^ between 0.72 − 311.65 ppm.

**Table 1:** Chemical and physical factors in the artificial saliva and toothpaste slurries.

**Group**	**Mean SL (SD)**	**Chemical factors**					**Physical factors**		
		**pH**	**[Ca** ^**2+**^ **] (mmol/l)**	**[PO**_**4**_^**3**−^ **] (µmol/l)**	**[F** ^−^ **] (ppm)**	**Presence of Sn** ^**2+**^	**%Weight of solid particles**	**Drop shape (mean angle, °)**	**Particle size (µm)**
Artificial Saliva	1.78 (0.44)	7.00	0.59	8.5 × 10^−3^	0.07	No	0.0	14.8	None
Colgate Caries Protection	2.24 (0.57)	6.89	0.23	4.8 × 10^−2^	39.66	No	45.5	17.3	<50
Sensodyne Repair and Protect	1.70 (0.32)	8.63	8.90	5.5 × 10^−1^	245.10	No	27.6	14.6	<50
elmex Sensitive Professional	2.30 (0.37)	8.75	0.29	6.0	62.09	No	40.8	19.5	<20
Sensodyne Rapid Relief	2.64 (0.39)	6.52	0.52	7.4 × 10^−4^	96.44	No	28.0	30.4	≥50
Blend-a-Med Pro Expert	3.99 (0.46)	5.57	0.18	3.0 × 10^−4^	311.10	Yes	34.7	8.6	≥50
Sensodyne Pronamel	2.24 (0.57)	7.03	0.09	7.2 × 10^−3^	311.65	No	25.3	29.7	<50
elmex Erosion Protection	2.21 (0.35)	4.70	0.62	8.4 × 10^−8^	253.20	Yes	26.9	26.7	<50
Candida Protect Professional	3.01 (0.40)	6.91	2.27	2.8 × 10^−2^	25.20	No	31.0	20.1	<50
Regenerate	3.60 (0.61)	9.02	0.08	66.1	0.72	No	34.7	7.5	≥50

The regression analyses are presented in Table 2. The pH and F^−^ concentration were not significantly associated with SL. All other factors were significantly associated with SL in the bivariate analysis and remained significant in the multivariate model. In the bivariate analysis, presence of Sn^2+^ and % weight of solid particles showed a positive association to SL, the direction of the association changed in the multivariate model. The multivariate analysis showed that lower SL was associated with higher concentrations of Ca^2+^ and PO_4_^3−^, presence of Sn^2+^, higher % weight of solid particles, smaller particle size, and lower wettability.

**Table 2:** Association between surface loss and the different chemical and physical factors in the toothpaste slurries.

Independent variable	Bivariate model*		Multivariate model^†^	
	Estimate ± SE	p-value	Estimate ± SE	p-value
Chemical factors				
pH	−0.063 ± 0.052	0.228		
Ca^2+^ concentration	−0.109 ± 0.025	<0.001	−0.247 ± 0.031	<0.001
PO_4_^3+^ concentration	0.017 ± 0.003	<0.001	−0.016 ± 0.004	<0.001
F^−^ concentration	<0.001	0.795		
Presence of Sn^2+^				
Not present^‡^	0		0	
Sn^2+^ present	0.662 ± 0.163	<0.001	−0.459 ± 0.155	0.003
Physical factors				
%weight of solid particles	0.024 ± 0.006	<0.001	−0.075 ± 0.014	<0.001
Drop shape (angle)	−0.041 ± 0.008	<0.001	−0.108 ± 0.013	<0.001
Particle Size				
No particles^‡^	0		0	
≤20 µm	0.513 ± 0.226	0.023	4.092 ± 0.644	<0.001
20 to 50 µm	0.499 ± 0.175	0.004	4.125 ± 0.592	<0.001
≥50 µm	1.629 ± 0.184	<0.001	4.554 ± 0.553	<0.001

*Analyses of each independent variable with the outcome (SL5) variable.

^†^Only variables with a p-value < 0.2 in the bivariate model were considered for the multivariate analysis, and the variables were only kept in the multivariate model if p-value < 0.05.

^‡^Reference category.

SE = Standard error of the estimate.

## Discussion

The present study shows that the claim of the toothpastes is not associated to the amount of SL observed, so we can accept our first hypothesis. All toothpastes caused different degrees of SL, and none were able to protect enamel from erosion and abrasion, confirming our second hypothesis. Our regression analyses show that different chemical and physical factors from the toothpastes are associated with SL, and we reject our third hypothesis.

The frequent contact of erosive substances with enamel results in a softening of the surface. The softened enamel surface is more prone to be lost when mechanical forces are applied^7^. In our study, we also evaluated the surface microhardness of the specimens after each erosive challenge and after brushing abrasion. The results are shown in Appendix 1 (supplementary file). As expected, enamel softening occurred after the erosive challenges, and the subsequent brushing abrasion procedure caused the partial removal of the softened layer. AS does not contain any abrasives, so the SL observed in this group can be explained by the mechanical action of the toothbrush itself^8^. Among the desensitizing and/or anti-erosive toothpastes tested, half were not significantly different from AS. So, we can suppose that they did not cause further SL other than that related to the toothbrush procedure. Moreover, these toothpastes may contain factors positively acting on enamel, protecting it against further erosion-abrasion challenges.

Remarkably, pH and fluoride were not associated to SL. It is important to bear in mind that all toothpastes tested in this study contain declared fluoride concentration varying from 1040 to 1450 ppm. Our values however, represent the free fluoride in the toothpaste slurries, which would be responsible for any action on enamel protection during this experiment. The difference between the fluoride concentration declared on the toothpaste tubes and our results can, therefore, be explained by the presence of artificial saliva in the slurries, which contain calcium and phosphate that could already react with the fluoride from the toothpastes, as well as by the dilution of the toothpastes in the slurries (1:2; w/w). Moreover, some of the toothpastes contain sodium monofluorphosphate (MFP) that requires enzymatic degradation to release fluoride, also explaining the lower concentration of free fluoride. The mode of action of fluoride in erosion prevention is mostly related to the formation of a calcium-fluoride-like (CaF_2_-like) layer on the enamel surface, which temporarily protects the underlying enamel against erosive substances^9,10^. The formation of CaF_2_-like material on the enamel surface is limited to the amount of free fluoride in the slurries and also dependent on a lower pH. Additionally, this layer is easily lost when in contact to erosive acids^11,12^. Lussi and Carvalho^13^ already discussed the limited protection of fluoride alone on ETW when applied with the goal of enhancing the formation of CaF_2_-like deposits. Our results, therefore, reflect the findings of other studies that did not show further enamel protection with monovalent fluoride compounds under enamel erosion/abrasion conditions and in a short experimental time^14,15^. However, it is worth mentioning that although having a limited action on enamel protection against ETW, the presence of fluoride in the toothpastes is still important, especially for caries prevention^16^.

In relation to the pH of the slurries, other studies have already shown no significant impact of pH on enamel protection^9,17^. One of the reasons for the lack of association of pH with SL could be its influence on chemical reactions. Different reactions need different pHs to occur. As an example, the tin-containing toothpastes are the ones with the lowest pH. One could expect this low pH to have a negative effect on SL, but the low pH is necessary for the mechanism of action of tin against SL^18^. So any negative influence the low pH would have on SL is, therefore, counteracted, and hopefully outperformed, by the positive influence of the active ingredients that need a low pH to react. Thus, our results did not show an association between pH and SL probably because the pH of each of the toothpastes is set to the level necessary for their ingredients to be active.

Curiously, when individually analysed, the presence of Sn^2+^ did not show a protective effect against SL. In fact, only two of the toothpastes tested contain tin, and they presented significantly different SL values. It is possible that the high SL from Blend-a-Med Pro Expert toothpaste led to a negative association in the bivariate regression. However, when the presence of Sn^2+^ was analysed with all other factors in the multivariate analysis, it led to a positive association, indicating a protective effect against SL. This means that the presence of Sn^2+^ alone should not be regarded as a protecting factor against SL. The protecting effect of Sn^2+^ is related to the interplay between chemical and physical factors that will also play a role on SL when abrasive forces are applied. This is in accordance with other studies that showed that the protecting action of tin is better observed when the toothpaste is applied as slurry (without brushing), whereas, when brushing occurs, the protecting effect is decreased^4–6^. Importantly, although the fluoride was not significantly associated to SL in the present study, its combination with tin has been shown to significantly enhance the protection of enamel against ETW^13,19,20^.

In the present study, higher concentrations of Ca^2+^ and PO_4_^3−^ in the slurries were also associated to less SL. Their effect is well understood on caries lesions, where these ions are retained and interact with enamel/plaque^21^. However, their role in ETW is still not fully understood and more studies are necessary to elucidate their mode of action regarding their presence in toothpastes.

Among the physical factors analysed, all variables were significantly associated to SL. The % weight of solid particles showed a negative association, which means that lower SL is expected when there are greater amounts of solid particles. Ganss *et al*.^6^ speculated that, in slurries with greater amount of abrasives, the particles can agglomerate and be taken away from the enamel surface, or that the particles become more densely packed within the grooves of the demineralized enamel hindering them from rolling over the enamel surface during the brushing movements. This means that greater amount of particles could not cause additional SL. Moreover, the presence of abrasives has been associated with SL up to a certain concentration, and greater amounts are related to lower SL^5,6^.

All the toothpastes analysed contain small particles (<20 µm), however, five of them also have big particles, up to 50 µm, and only three have particles bigger than 50 µm. In fact, the two toothpastes with the highest SL contain big particles. This explains our multivariate analysis, that bigger particles are associated to more SL. On the other hand, in the study from Ganss *et al*.^5^, particle size seemed to have no impact on SL. This contradiction could be due to the different model used in their study. We analysed initial ETW (our model was based on a total of 15 min of erosion and 125 s of abrasion), while Ganss *et al*.^5^ used a more severe challenge (total of 120 min of erosion and 300 s of abrasion). Thus, we speculate that the presence of bigger particle sizes is associated to an increased SL for initial ETW lesions, but this association may diminish as ETW challenges become more severe.

Another physical factor associated to SL is the wettability of the slurry. Our results showed that lower wettability lead to lower SL. Observing Table 1, the two toothpastes with higher SL (Blend-a-Med Pro Expert and Regenerate) are the ones that presented smaller angles in the drop shape analysis, in other words, higher wettability. This means that the slurry spreads more easily over the enamel surface. This finding is a little surprising since we would expect that higher wettability would allow more contact of the active ingredients with the enamel surface. Likewise, it would also spread the abrasives over a greater area of the enamel surface, allowing their contact with a larger area of enamel. This may contribute to a greater removal of the softened enamel layer. The two toothpastes with higher wettability are also the ones with bigger particles, so we speculate that the association between big particles and higher wettability causes more SL. However, studies analysing the wettability of toothpastes are limited, and further experiments are still necessary.

One limitation of our study is that we did not evaluate the relative enamel abrasivity (REA) and/or relative dentin abrasivity (RDA) of the toothpastes. These values are dependent on the amount and type of abrasives present in the toothpaste^6,22,23^. However, for the regression analysis, the variables should be independent from each other. For our analyses, we considered the abrasive particle size and amount of solid particles, whereas REA and RDA values could lead to collinearity. Moreover, recent studies have shown minor relevance of REA and RDA values for SL under erosive conditions^4,5^. Another limitation of our study is that the chemical and physical factors of the toothpaste slurries were analysed in an *in vitro* model, using artificial saliva for specimen incubation and for the preparation of the slurries. This means that we introduced in our slurries a dilution factor to the toothpastes, as well as other ion-components that would interact with the ingredients from the toothpastes. *In vivo*, the dilution and reaction of the toothpastes may be different, and this will depend on the patients’ characteristics and habits. Moreover, the buffer capacity of the toothpastes were not analysed in this study. In any case, because our specimens were washed with DDW after each erosive challenge, the reaction between the acid and the enamel is quenched, and the demineralization is halted. This minimizes the possible buffering effect the toothpastes would have on the acid. However, *in vivo*, the buffering capacity of the toothpastes may play a role in acid neutralization, and this factor should be considered.

The Knoop indentations were used to calculating the enamel surface loss. This is a well-known technique for measuring initial enamel erosion^19,24^, and it allows the analysis of the loss related to the abrasion procedure^8^. We performed the indentation after the erosive challenge and then re-measured the indentation after the abrasion, making possible to quantify the amount of softened enamel removed by the abrasion procedure. Another common method used to measure surface loss is the profilometer^5,25,26^. However, a recent study showed that, although the different methods usually present different numerical values of surface loss measurements, both the indentation method and the profilometry have shown good correlation and either can be used in surface loss studies^27^.

We can conclude from this study that enamel surface loss occurred to different extents, regardless of the claim of the toothpastes (desensitizing and/or anti-erosive). Although some of the toothpastes showed lower surface loss, none of them was able to fully protect the enamel against ETW. From the chemical and physical factors analysed in the toothpaste slurries, only pH and F^−^ were not significantly associated to surface loss. Lower surface loss was associated with the following factors from the toothpaste slurries: higher concentration of Ca^2+^ and PO_4_^3−^, presence of Sn^2+^, higher % weight of solid particles, smaller particle size, and lower wettability.

## Materials and Methods

### Specimen preparation

We prepared 150 enamel specimens from sound human premolar surfaces, which were obtained from a pooled bio-bank and had been stored in 2% chloramine T trihydrate solution. The patients were previously informed about the possibility of using their teeth for research purposes, and consent was obtained. The experiment was carried out in accordance with the approved guidelines and regulations of the local ethics committee (Kantonale Ethikkommission: KEK), which categorized the teeth as “irreversibly anonymized” because they had been pooled, so no previous approval from the committee was necessary.

The premolars were embedded in acrylic resin (Paladur, Heraeus Kulzer GmbH, Hanau, Germany) and the specimens were serially abraded under constant tap water cooling (LabPol 21, Struers, Ballerup, Denmark) with silicon carbide paper discs of decreasing grain size (18.3 µm to 5 µm), leaving a smooth flat enamel surface. This procedure removed the outer 200 μm layer of the enamel. A final polishing was made for 60 s (LabPol 6, Struers), under constant cooling, using a 3 μm grain diamond paste (DP-Stick P, Struers). Then, the specimens were stored in a mineral solution (1.5 mmol/l CaCl_2_, 1.0 mmol/l KH_2_PO_4_, 50 mmol/l NaCl, pH = 7.0)^28^ until the beginning of the experiment, when the specimens were submitted to a further 60 s of polishing with 1 μm grain diamond paste under constant cooling^19^.

### Experimental groups

Sample size calculation was performed with the program G*Power (version 3.1.9.2). Considering an effect size of 1.6, Type I error α = 0.05, and a power (1 − β) of 0.8, we have calculated a sample size of 8 specimens per group (actual power 0.83; t = 2.16). In this study, we used 15 specimens per group.

The specimens were randomly distributed into 10 groups, according to the toothpastes tested (Table 3). As controls, artificial saliva (the same as used for slurry preparation) and a regular fluoridated toothpaste (Colgate Caries Protection) were used. Eight other toothpastes were tested, where the main claim was either desensitizing (4 toothpastes) or anti-erosive (4 toothpastes).

**Table 3:** Details of the manufacturer, claim and active ingredients of the toothpastes.

**Group**	**Manufacturer; country of acquisition**	**Claim**	**Active Ingredients**	**Lot**
Artificial Saliva		Negative control		
Colgate Caries Protection	Colgate-Palmolive; Switzerland	Positive control	1450 ppm F (1000 ppm as MFP and 450 ppm as NaF)	8018PL111
Sensodyne Repair and Protect	GlaxoSmithKline; France	Desensitizing	1450 ppm F (as NaF) NovaMin^®^ (Calcium Sodium Phosphosilicate)	016A
elmex Sensitive Professional	Colgate-GABA; Switzerland	Desensitizing	1450 ppm F (as MFP) Arginine	5357PL111
Sensodyne Rapid Relief	GlaxoSmithKline; Switzerland	Desensitizing	1040 ppm F (as NaF) Strontium Acetate	50962kWA
Blend-a-Med (Oral-B) Pro Expert	Procter and Gamble; Switzerland	Desensitizing	1450 ppm F (1100 ppm as SnF_2_ and 350 ppm as NaF)	6036GG81
Sensodyne Pronamel	GlaxoSmithKline; Switzerland	Anti-erosive	1450 ppm F (as NaF) Potassium Nitrate	60482KWC
elmex Erosion Protection	Colgate-GABA; Switzerland	Anti-erosive	1400 ppm F (as AmF and NaF; concentrations not specified) 3500 ppm tin (as SnCl_2_) Chitosan (0.5%)	4311PL1031
Candida Protect Professional	Migros; Switzerland	Anti-erosive	1450 ppm F (as MFP) Oligopeptide 104	94867–03
Regenerate	Unilever; France	Anti-erosive	1450 ppm F (as MFP) Calcium Silicate Sodium Phosphate	530880B

Artificial saliva [1.25 mM Ca(NO_3_)_2_·4H_2_O, 0.90 mM KH_2_PO_4_, 129.91 mM KCl, 59.93 mM Tris buffer and 2.2 g/l porcine gastric mucine; pH 7.4]^29^ was prepared weekly in the laboratory and stored at −20 °C. Before the beginning of each cycle, a fresh aliquot of artificial saliva was thawed and used to incubate the specimens and to prepare the toothpaste slurries. The preparation of the toothpaste slurries was performed daily, right before the toothbrush abrasion, by mixing 25 g of the toothpaste with 50 g of artificial saliva^19^.

### Erosion-abrasion cycles

The specimens were submitted to an erosion-abrasion cycling model of five days, with one cycle/day. Each cycle consisted of individually incubating the specimen in artificial saliva in a shaking water bath (60 min, 37 °C, 70 rpm, travel path 50 mm), followed by erosive demineralization in 1% citric acid (pH 3.6, 3 min, 25 °C, 70 rpm, travel path 50 mm). After each immersion, the specimens were rinsed with tap water for 15 s and distilled deionized water (DDW) for 5 s, then dried with oil-free air for 5 s. Subsequently, each specimen was mounted in a container of an automatic brushing machine (Zahnbürstmaschine, Syndicad Ingenieurbüro, Munich, Germany), which received freshly made toothpaste slurry or artificial saliva (AS), according to the experimental groups. The specimens were immersed in the slurry or AS for 2 min (room temperature), during this time they were brushed for 25 s (50 toothbrush strokes, ca. 2 N, 120 strokes/min, travel path 40 mm, 40 mm/s). The specimens were then rinsed for 20 s in DDW and dried with oil-free air for 5 s. The specimens were constantly kept in a humid chamber, at room temperature, while not in use.

### Enamel surface loss (SL) calculation

The enamel surface loss was calculated according to previous studies from our group^19,24,30^. After each erosion challenge, six Knoop indentations were made on the enamel surface, with a load of 200 g and a dwell time of 10 s (UHL VMHT Microhardness Tester). The lengths of the indentations were measured before and immediately after the toothbrush abrasion. Using the length values (L), the indentation depth (D) was calculated according to the equation: D = L/2·tan α, where α is a constant parameter of the diamond indenter (α = 3.75°). The difference in depth (µm) of the same indentation before and after the toothbrush abrasion was calculated and this value represents the amount of enamel abraded away from the surface (SL). The average value of the six indentations was considered as the SL after the abrasive challenge for each specimen, after each experimental cycle. After 5 cycles, the total SL (SL5) was calculated by taking a sum of the values of all cycles.

### Chemical analyses of the toothpaste slurries

As chemical factors of the toothpaste slurries, we considered the pH, the presence of tin (Sn^2+^, according to the description in the toothpaste tube by the manufacturer) and, the concentrations of calcium (Ca^2+^), phosphate (PO_4_^3−^) and fluoride (F^−^) in the toothpastes slurries.

The pH of the freshly prepared slurries was measured at room temperature using a pHmeter (691 pH Meter, Metrohm, Switzerland). For the Ca^2+^, PO_4_^3−^ and F^−^ concentrations measurements, the toothpaste slurries were centrifuged (20 min, 3000 rcf, 25 °C) and properties of the supernatants were measured. Total Ca^2+^ concentration was measured with an atomic absorption spectrometer with an air/acetylene flame (AAnalyst400, Perkin-Elmer, USA). Total inorganic phosphates (P_i_) concentration was analysed by the ammonium molybdate method of Chen *et al*.^31^ with a spectrometer (Lambda 2 UV-VIS, Perkin-Elmer, Germany). From the total P_i_ concentration, PO_4_^3−^ concentration was calculated, considering the pH of the slurries, using the following equation:

$$[P{O}_{4}^{3-}]=(\frac{{K}_{a1}\,{K}_{a2}\,{K}_{a3}}{{[{H}_{3}{O}^{+}]}^{3}+{[{H}_{3}{O}^{+}]}^{2}{K}_{a1}+[{H}_{3}{O}^{+}]{K}_{a1}{K}_{a2}+{K}_{a1}{K}_{a2}{K}_{a3}})[{P}_{i}]$$

Where the different *K*_*a*_ values are the dissociation constants of phosphoric acid (*K*_*a*1_ = 0.0071; *K*_*a*2_ = 6.3 × 10^−8^; *K*_*a*3_ = 4.5 × 10^−13^).

Free F^−^ concentration was determined using a F^–^specific electrode (Orion 960900, Boston, MA, USA). The slurries were incubated for 60 min at 37 °C before centrifuging and adding total ionic strength adjustment buffer (TISAB III, 1:1 ratio). The concentration of Ca^2+^, PO_4_^3−^ and F^−^ are expressed in mmol/l, µmol/l and ppm, respectively. All analyses were made in duplicate.

### Physical analyses of the toothpaste slurries

As physical factors of the toothpaste slurries, we analysed the weight of the solid particles, the particle size, and the slurry wettability. For both the weight of solid particles and particle size analyses (adapted from Ganss *et al*.^5^), the toothpastes slurries were prepared using deionized water instead of artificial saliva. For the weight of solid particles, 40 ml of toothpaste slurries were weighed and later centrifuged (20 min, 3000 rcf, 25 °C). The supernatant was carefully removed and the remaining solids were left to dry at 40 °C for 10 days. Subsequently, the dried solid particles were weighed, and the percentage weight of solid particles was calculated in relation to the total weight of toothpaste slurry (% weight of solid particles). For the particle size analyses, the dried solids were pulverized using mortar and pestle, fixed on aluminium stubs with self-adhesive film, and sputter-coated with gold palladium. The particles were analysed using scanning electron microscopy (JSM-6010PLUS/LV SEM, JEOL, Tokyo, Japan). The particles size was determined by analysing three images (magnification of 300x; at 14 kV), using the measurement tool of the software of the SEM. The particles sizes were described according to size ranges: <20 µm; <50 µm and ≥50 µm.

For the wettability analysis, we used a drop shape contact angle device (Drop Shape Analysis System DSA 10 MK2, Krüss, Germany; needle Ø = 1.1 mm, drop volume 1 µl). For that, the slurries were passed 5 times through a sieve (300 µm mesh) to remove the biggest particles and allow a drop to form from the needle. A droplet was placed on three different locations of a flat and polished enamel surface. The average of the three contact angles was calculated for each group, and the results are expressed as contact angle of the drop with the surface. The contact angle of the drop has an inverse relation with the wettability results; in other words, larger angles imply lower wettability, and vice-versa.

### Statistical analyses

Data of the total SL was analysed using R software, version 3.3.3. In order to quantify the effects of “group” and “time” factors on the outcome, non-parametric repeated measures ANOVA was performed^32^. Post-hoc analyses were made using Mann-Whitney-Wilcoxon tests and Bonferroni-Holm corrections for multiple testing. Statistical significant level was set at 0.05.

For the analyses of the association of chemical and physical factors with the total SL, general linear models were used. Initially, bivariate regression analyses were performed considering each factor and SL. The bivariate analyses, however, consider each independent variable individually; they do not take into consideration the interplay of the different chemical and physical factors that influence together the total SL. This interplay was analysed with multivariate regression analysis. For this analysis, we selected the factors that presented p-values < 0.20 in the bivariate models. Then, we included these variables in the regression model using a backward stepwise approach. Variables with a p-value < 0.05 were retained in the final regression model. The regression analyses were performed using IBM SPSS Statistics version 22.

## Electronic supplementary material

Appendix A
