# Supplementary material for: Chemical and physical factors of desensitizing and/or anti-erosive toothpastes associated with lower erosive tooth wear
Source: Sci Rep. 2017 Dec 20;7:17909. doi: 10.1038/s41598-017-18154-8 (PMC5738370; doi:10.1038/s41598-017-18154-8)
Supplement: Supplementary file 1 — Appendix A [file 41598_2017_18154_MOESM1_ESM.doc]

**Chemical and physical factors of desensitizing and/or anti-erosive toothpastes associated with lower erosive tooth wear**

**Authors:** Samira Helena João-Souza, Adrian Lussi, Tommy Baumann, Taís Scaramucci, Ana Cecília Corrêa Aranha, Thiago Saads Carvalho.

**Appendix A**

Besides analysing the enamel surface loss (SL), we also analysed the surface microhardness (SMH) change throughout the experimental cycles. This appendix describes the methods, analyses and results for SMH.

**Material and methods**

*Surface microhardness (SMH) measurement*

For the SMH measurements, we used a Knoop microhardness tester (UHL VMHT Microhardness Tester, UHL technische Mikroskopie GmbH & Co. KG, Asslar, Germany). For each SMH measurement, six indentations of 10 g load with a dwell time of 10 s were performed on the enamel surface in 25 μm intervals. The average value from the six indentations was used to calculate the SMH. This analysis was realized at the beginning of the experiment (SMH-Initial) and after every erosive challenge (SMH-ER-i) and toothbrush abrasion (SMH-AB-i), where i represents the experimental cycle (1 – 5).

The relative SMH (rSMH) after the last erosive challenge (ER-5) was calculated using the following formula:

rSMH = 100 x (SMH-ER-5/SMH-Initial)

The evolution of SMH across the cycles was calculated with the same formula described above. However, instead of SMH-ER-5, we used SMH-ER-i or SMH-AB-i, according to each experimental time.

rSMH = 100 x (SMH-ER-i/SMH-Initial)

rSMH = 100 x (SMH-AB-i/SMH-Initial)

*Statistical analyses*

The rSMH after the last erosive challenge (SMR-ER-5) was analysed using repeated measures ANOVA (Brunner E, Domhof S and Langer F. Nonparametric Analysis of Longitudinal Data in Factorial Experiments, 2002, New York: Wiley). Post-hoc analyses were performed using Mann-Whitney-Wilcoxon tests and Bonferroni-Holm corrections for multiple testing. Statistical significant level was set at 0.05. The statistical analyses were calculated using R 3.3.3.

For the analyses of the association of chemical and physical factors of the toothpaste slurries with the rSMH after the last erosive challenge (ER-5), general linear models were used as described in the manuscript.

**Results**

Figures I and II show the rSMH for the different experimental steps at each cycle for desensitizing (Figure I) and/or anti-erosive (Figure II) toothpastes. After the first acid challenge, all groups showed decreased rSMH, in the same level, due to the effect of the citric acid on the enamel surface. When the toothbrush abrasion was performed, the rSMH increased for all groups, in different levels. This pattern of SMH decrease after erosion and increase after abrasion was maintained throughout the experiment. After the successive erosion and abrasion challenges, we see that the rSMH pattern varies between the groups. Already at AB-1, we notice that the rSMH increases to different degrees for different toothpastes. This means that no group could recover the initial microhardness, on the contrary, rSMH increased because of the removal of the softened enamel. This is observed in the results on the main manuscript.

After the last erosive challenge, as expected, all groups presented decreased rSMH (Figure III), which is related to the acid effect on the enamel surface. The Artificial Saliva (AS) demonstrated the lowest value of rSMH. This is probably due to the absence of abrasive particles in AS, which has a minimal impact on the removal of the softened enamel. So, the remaining softened enamel layer after the brushing abrasion will probably increase in depth after the subsequent erosive challenges. This can be observed in the Figures I and II, where the rSMH of AS progressively decreases. Conversely, the toothpastes presented higher rSMH after the last erosive challenge when compared to the AS. This is explained by the presence of abrasives in the toothpastes, which acts on the increased removal of the softened enamel layer during brushing. Consequently, the subsequent acid challenge will act on a harder enamel layer. As the toothpastes remove the softened enamel to different degrees, and the following acid challenge forms a new softened layer, the groups do not show much difference in rSMH-ER-5, as observed in Figure III. The difference on the effect of the toothpastes can be better seen in the surface loss results (main manuscript).

Individually analyzing the chemical and physical factors of the toothpastes slurries, PO_4_^3-^ and F^-^ concentration, presence of Sn^2+^, %weight of solid particles and particle sizes showed significant association to rSMH (Table I), but only in bivariate models. However, these factors lost significance when analyzed together in the multivariate regression (p>0.05), and a multivariate model could not be presented.

Figure I. Relative surface microhardness (rSMH) throughout the experiment for desensitizing toothpastes.


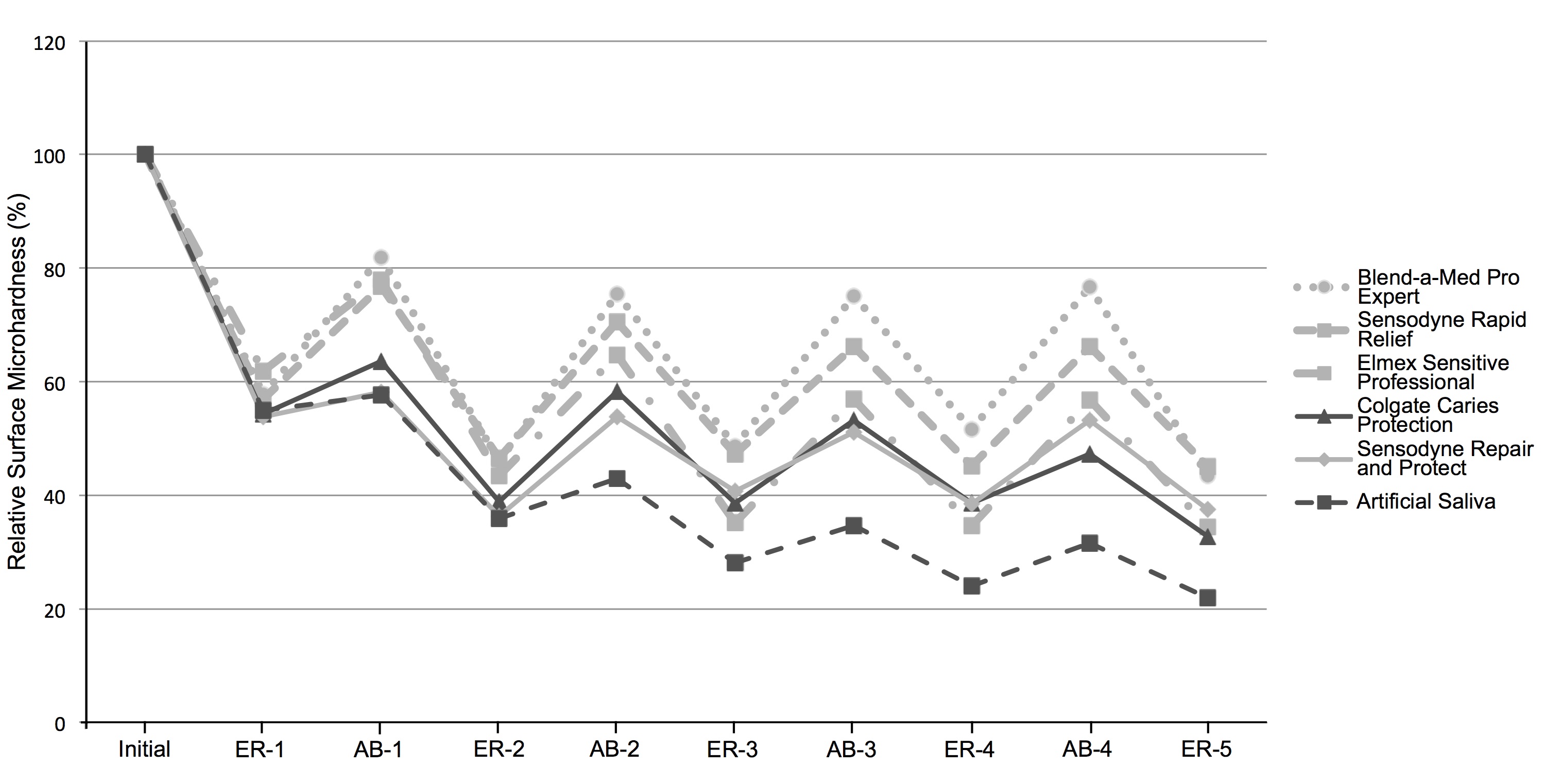


Figure II. Relative surface microhardness (rSMH) throughout the experiment for anti-erosive toothpastes.


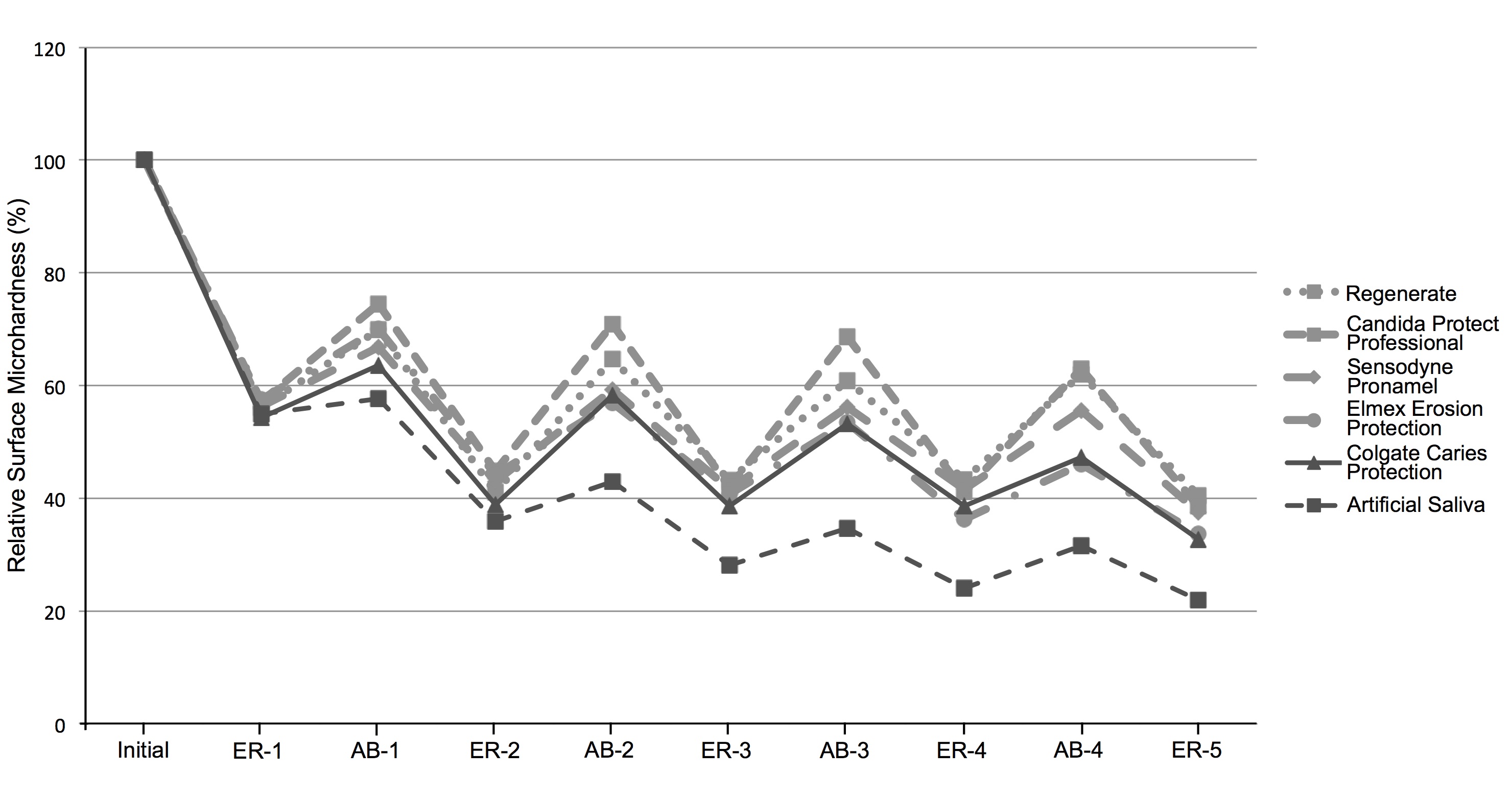


Figure III. Relative surface microhardness (rSMH) after the last erosion challenge (ER-5) and statistical analysis results: light grey boxes – control groups, medium grey boxes – desensitizing claim; dark grey boxes – anti-erosive claim. Different letters denote significant differences between the groups.


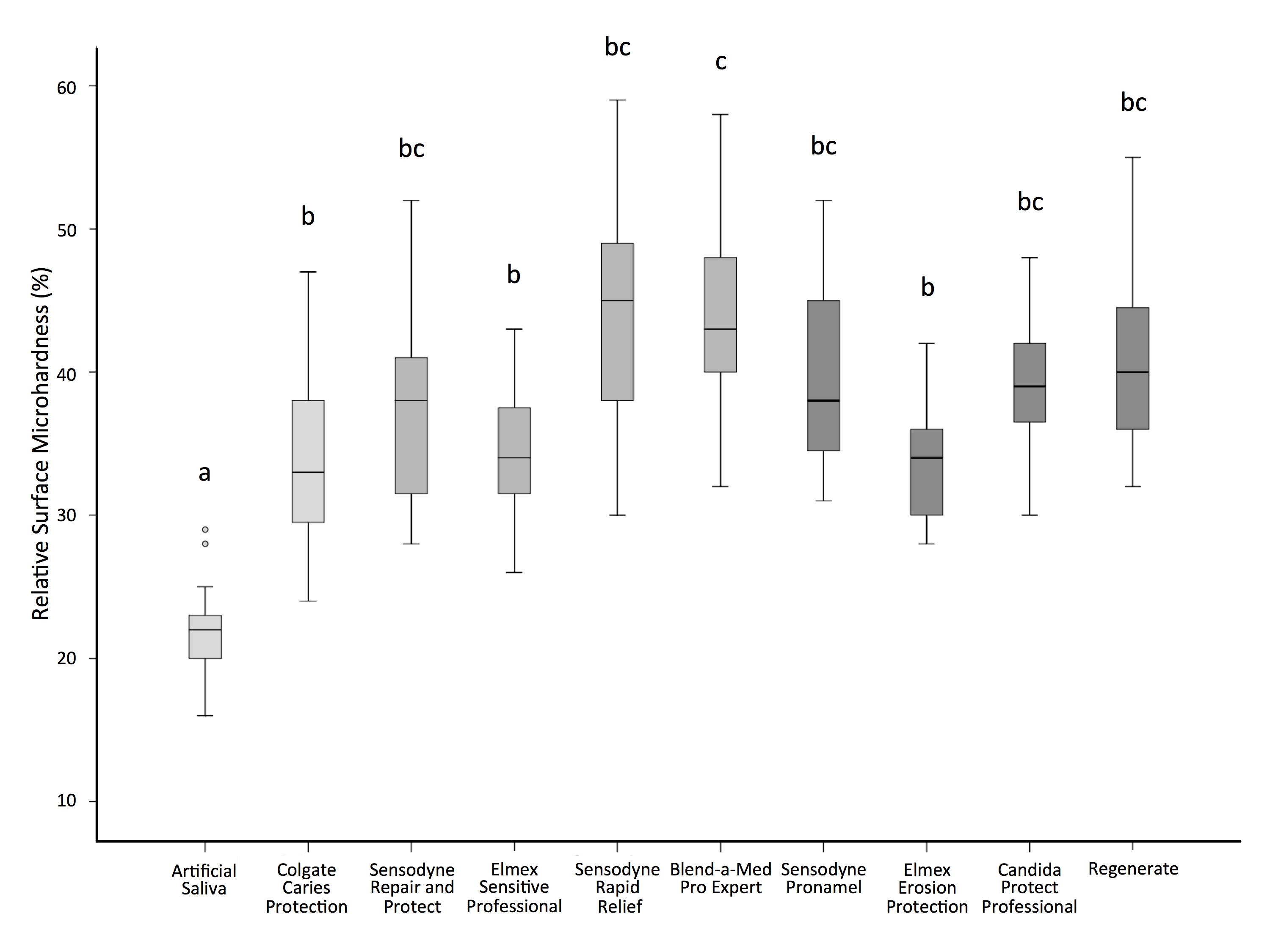


| Independent variable | Bivariate model* | |
| --- | --- | --- |
|  | Estimate ± SE | p-value |
| Chemical factors |  |  |
| pH | -0.140 ± 0.537 | 0.794 |
| Ca^2+^ concentration | 0.021 ± 0.274 | 0.938 |
| PO_4_^3+^ concentration | 0.065 ± 0.036 | 0.068 |
| F^-^ concentration | 0.019 ± 0.006 | 0.001 |
| Presence of Sn^2+^ |  |  |
| Not present | 0 |  |
| Sn^2+^ present | 2.675 ± 1.752 | 0.127 |
| Physical factors |  |  |
| %Weight of solid particles | 0.312 ± 0.056 | <0.001 |
| Drop shape (angle) | 0.043 ± 0.092 | 0.643 |
| Particle Size |  |  |
| No particles | 0 |  |
| ≤ 20 µm | 12.400 ± 2.294 | <0.001 |
| 20 to 50 µm | 14.760 ± 1.777 | <0.001 |
| ≥ 50 µm | 21.356 ± 1.873 | <0.001 |
| * Analyses each independent variable with the outcome (rSMH) variable;  ^‡^ Reference category;  SE = Standard error of the estimate. | | |

Table I. Association between relative surface microhardness (rSMH-ER-5) and chemical and physical factors from the toothpastes slurries.

NOTE: When analysed individually (in the bivariate model), some variables were significantly associated with rSMH, but they all lost significance in a multivariate analysis (p>0.05). Therefore, the multivariate model was not built.
